# Supplementary material for: Association between obstructive sleep apnea risk and atherosclerosis: A nationwide cross-sectional study in the Korean population
Source: PLoS One. 2025 May 8;20(5):e0322897. doi: 10.1371/journal.pone.0322897 (PMC12061421; doi:10.1371/journal.pone.0322897)

PLOS ONE

Association between Obstructive Sleep Apnea Risk and Atherosclerosis: A Nationwide Cross-sectional Study in the Korean Population

--Manuscript Draft--

Manuscript Number:	PONE-D-24-46140
Article Type:	Research Article
Full Title:	Association between Obstructive Sleep Apnea Risk and Atherosclerosis: A Nationwide Cross-sectional Study in the Korean Population
Short Title:	OSA and Atherogenic Risk in Koreans
Corresponding Author:	Seung Min Chung<br>Yeungnam University School of Medicine and College of Medicine<br>Daegu, KOREA, REPUBLIC OF
Keywords:	sleep apnea, obstructive; atherosclerosis; Diabetes Mellitus
Abstract:	Objectives<br><br>Obstructive sleep apnea (OSA) increases the risk of all-cause and cardiovascular mortality. This study aimed to investigate the association between OSA and atherogenic risk in the Koreans.<br><br>Methods<br><br>Data from 8,158 participants (mean age, 57.9 ± 11.7; male/female, 1:1.4) obtained from the Korea National Health and Nutrition Examination Survey between 2019 and 2021. OSA risk was screened using the STOP-BANG score, and atherogenic risk was measured using the atherogenic index of plasma (AIP). Logistic regression was used to evaluate the association between the STOP-BANG scores and high AIP and subgroups according to the presence of diabetes.<br><br>Results<br><br>The proportions of individuals with atherogenic risk (AIP >0.24) were 13.7%, 27.6%, and 34.7% in the low-, intermediate-, and high-OSA risk groups (p <0.001). After adjustment, individuals with intermediate and high OSA risk had 1.35 (95% confidence interval [CI], 1.16–1.58; p <0.001) and 1.32 (95% CI, 1.08–1.61; p = 0.006) times higher odds of having atherogenic risk than those with low OSA risk. Among patients without diabetes, high OSA risk was not an independent factor affecting atherogenic risk (hazard ratio [HR], 1.17; 95% CI, 0.93–1.47). However, among patients with diabetes, compared with those with low OSA risk, those with intermediate (HR, 1.51; 95% CI, 1.05–2.19) and high OSA risk (HR, 1.58; 95% CI, 1.02–2.46) had significantly increased atherogenic risk.<br><br>Conclusion<br><br>OSA is linked to increased atherogenic risk in the Koreans, especially in individuals with diabetes, thus highlighting the importance of routine OSA screening to manage and reduce cardiovascular risks.
Order of Authors:	Il Rae Park
	Yong Geun Chung
	Seung Min Chung
	Jun Sung Moon
	Ji Sung Yoon
	Kyu Chang Won
Additional Information:	

Powered by Editorial Manager® and ProduXion Manager® from Aries Systems Corporation

Question	Response
**Financial Disclosure**<br><br>Enter a financial disclosure statement that describes the sources of funding for the work included in this submission. Review the submission guidelines for detailed requirements. View published research articles from *PLOS ONE* for specific examples.<br><br>This statement is required for submission and **will appear in the published article** if the submission is accepted. Please make sure it is accurate.<br><br>**Funded studies**<br>Enter a statement with the following details:<br>• Initials of the authors who received each award<br>• Grant numbers awarded to each author<br>• The full name of each funder<br>• URL of each funder website<br>• Did the sponsors or funders play any role in the study design, data collection and analysis, decision to publish, or preparation of the manuscript?<br><br>Did you receive funding for this work?	The author(s) received no specific funding for this work.
**Competing Interests**<br><br>Use the instructions below to enter a competing interest statement for this submission. On behalf of all authors, disclose any competing interests that could be perceived to bias this work—acknowledging all financial support and any other relevant financial or non-financial competing interests.<br><br>This statement is required for submission and **will appear in the published article** if the submission is accepted. Please make sure it is accurate and that any funding sources listed in your Funding Information later in the submission form are also	The authors have declared that no competing interests exist.

Powered by Editorial Manager® and ProduXion Manager® from Aries Systems Corporation

declared in your Financial Disclosure statement.<br><br>View published research articles from *PLOS ONE* for specific examples.<br><br>**NO authors have competing interests**<br><br>Enter: *The authors have declared that no competing interests exist.*<br><br>**Authors with competing interests**<br><br>Enter competing interest details beginning with this statement:<br><br>*I have read the journal's policy and the authors of this manuscript have the following competing interests: [insert competing interests here]*<br><br>* typeset	
**Ethics Statement**<br><br>Enter an ethics statement for this submission. This statement is required if the study involved:<br><br>• Human participants<br>• Human specimens or tissue<br>• Vertebrate animals or cephalopods<br>• Vertebrate embryos or tissues<br>• Field research<br><br>Write "N/A" if the submission does not require an ethics statement.<br><br>General guidance is provided below. Consult the submission guidelines for detailed instructions. **Make sure that all information entered here is included in the Methods section of the manuscript.**	This trial was approved by institutional review board of Yeungnam University Hospital (2024-07-034).

Powered by Editorial Manager® and ProduXion Manager® from Aries Systems Corporation

**Format for specific study types**<br><br>**Human Subject Research (involving human participants and/or tissue)**<br>• Give the name of the institutional review board or ethics committee that approved the study<br>• Include the approval number and/or a statement indicating approval of this research<br>• Indicate the form of consent obtained (written/oral) or the reason that consent was not obtained (e.g. the data were analyzed anonymously)<br><br>**Animal Research (involving vertebrate animals, embryos or tissues)**<br>• Provide the name of the Institutional Animal Care and Use Committee (IACUC) or other relevant ethics board that reviewed the study protocol, and indicate whether they approved this research or granted a formal waiver of ethical approval<br>• Include an approval number if one was obtained<br>• If the study involved *non-human primates*, add *additional details* about animal welfare and steps taken to ameliorate suffering<br>• If anesthesia, euthanasia, or any kind of animal sacrifice is part of the study, include briefly which substances and/or methods were applied<br><br>**Field Research**<br><br>Include the following details if this study involves the collection of plant, animal, or other materials from a natural setting:<br>• Field permit number<br>• Name of the institution or relevant body that granted permission	
**Data Availability**<br><br>Authors are required to make all data underlying the findings described fully available, without restriction, and from the time of publication. PLOS allows rare exceptions to address legal and ethical concerns. See the PLOS Data Policy and FAQ for detailed information.	Yes - all data are fully available without restriction

Powered by Editorial Manager® and ProduXion Manager® from Aries Systems Corporation

A Data Availability Statement describing where the data can be found is required at submission. Your answers to this question constitute the Data Availability Statement and **will be published in the article**, if accepted.<br><br>**Important:** Stating 'data available on request from the author' is not sufficient. If your data are only available upon request, select 'No' for the first question and explain your exceptional situation in the text box.<br><br>Do the authors confirm that all data underlying the findings described in their manuscript are fully available without restriction?	
**Describe where the data may be found in full sentences. If you are copying our sample text, replace any instances of XXX with the appropriate details.**<br><br>• If the data are **held or will be held in a public repository**, include URLs, accession numbers or DOIs. If this information will only be available after acceptance, indicate this by ticking the box below. For example: *All XXX files are available from the XXX database (accession number(s) XXX, XXX.).*<br>• If the data are all contained **within the manuscript and/or Supporting Information files**, enter the following: *All relevant data are within the manuscript and its Supporting Information files.*<br>• If neither of these applies but you are able to provide **details of access elsewhere**, with or without limitations, please do so. For example:<br><br>*Data cannot be shared publicly because of [XXX]. Data are available from the XXX Institutional Data Access / Ethics Committee (contact via XXX) for researchers who meet the criteria for access to confidential data.*<br><br>*The data underlying the results presented in the study are available from (include the name of the third party*	The Data from Korea Disease Control and Prevention Agency. The Eighth Korea National Health and Nutrition Examination Survey (KNHANES VIII-3). 2021 https://www.data.go.kr/data/15076556/fileData.do#

Powered by Editorial Manager® and ProduXion Manager® from Aries Systems Corporation

and contact information or URL).<br>• This text is appropriate if the data are owned by a third party and authors do not have permission to share the data.<br><br>* typeset	
Additional data availability information:	

Manuscript
Click here to access/download;Manuscript;Plosone 241022.doc

Association between Obstructive Sleep Apnea Risk and Atherosclerosis: A Nationwide Cross-sectional Study in the Korean Population

Il Rae Park[1¶], Yong Geun Chung[1], Seung Min Chung[1], Jun Sung Moon[1], Ji Sung Yoon[1], Kyu Chang Won[1]

[1]Department of Internal Medicine, Yeungnam University College of Medicine, Daegu

***Corresponding author:** Seung Min Chung

E-mail: smchung@ynu.ac.kr (SMC)

Abstract

Objectives

Obstructive sleep apnea (OSA) increases the risk of all-cause and cardiovascular mortality. This study aimed to investigate the association between OSA and atherogenic risk in the Koreans.

Methods

Data from 8,158 participants (mean age, 57.9 ± 11.7; male/female, 1:1.4) obtained from the Korea National Health and Nutrition Examination Survey between 2019 and 2021. OSA risk was screened using the STOP-BANG score, and atherogenic risk was measured using the atherogenic index of plasma (AIP). Logistic regression was used to evaluate the association between the STOP-BANG scores and high AIP and subgroups according to the presence of diabetes.

Results

The proportions of individuals with atherogenic risk (AIP >0.24) were 13.7%, 27.6%, and 34.7% in the low-, intermediate-, and high-OSA risk groups (p <0.001). After adjustment, individuals with intermediate and high OSA risk had 1.35 (95% confidence interval [CI], 1.16–1.58; p <0.001) and 1.32 (95% CI, 1.08–1.61; p = 0.006) times higher odds of having atherogenic risk than those with low OSA risk. Among patients without diabetes, high OSA risk was not an independent factor affecting atherogenic risk (hazard ratio [HR], 1.17; 95% CI, 0.93–1.47). However, among patients with diabetes, compared with those with low OSA risk, those with intermediate (HR, 1.51;

95% CI, 1.05–2.19) and high OSA risk (HR, 1.58; 95% CI, 1.02–2.46) had significantly increased atherogenic risk.

Conclusion

OSA is linked to increased atherogenic risk in the Koreans, especially in individuals with diabetes, thus highlighting the importance of routine OSA screening to manage and reduce cardiovascular risks.

Keywords: sleep apnea, obstructive; atherosclerosis; diabetes mellitus

Introduction

Obstructive sleep apnea (OSA) is characterized by recurrent partial or total obstruction of the upper airway during sleep that leads to apnea. OSA is a widespread sleep disorder, affecting approximately 936 million adults aged 30 to 69 years worldwide [1]. Furthermore, with the increasing prevalence of obesity and metabolic syndrome, the incidence of OSA is increasing.

OSA is associated with cardiovascular diseases (CVDs), cerebrovascular diseases, cognitive disorders, type 2 diabetes, and metabolic syndrome [2]. OSA shares similar pathophysiological features with metabolic disorders. The intermittent hypoxia during apnea, which is caused by upper airway obstruction, leads to tissue hypoxia, triggering a series of adverse physiological responses. These include increased sympathetic nervous system activity, oxidative stress, and systemic inflammation, which all contribute to coronary atherosclerosis, insulin resistance, and other metabolic dysfunctions [3]. Consequently, OSA is a significant independent risk factor for CVD [3,4]. A previous meta-analysis of 16 studies involving 24,308 individuals showed that severe OSA increases all-cause and cardiovascular mortality [5].

However, despite the risk associated with the disorder, it is frequently underdiagnosed owing to its occurrence during sleep, which limits patient awareness [6]. The gold standard for diagnosing OSA is polysomnography. However, polysomnography is complex, expensive, and lengthy, limiting its feasibility for widespread use [7]. The STOP-BANG score, created in 2008, is a simple and practical tool that assesses OSA risk through a questionnaire [8]. The questionnaire includes parameters such as snoring, daytime tiredness, observed apnea during sleeping,

hypertension, body mass index (BMI) >35 kg/m^2, age >50 years, neck circumference >40 cm, and male sex [8]. A prospective study involving 435 hospitalized patients showed that a STOP-BANG score ≥5 is an independent risk factor for cardiovascular mortality [9]. However, OSA risk is rarely assessed in diabetes care settings.

The atherogenic index of plasma (AIP) can be easily calculated based on lipid profile. It is a well-known marker related to insulin resistance, type 2 diabetes, and CVD and is considered to have better predictive accuracy than individual lipid profiles or glucose alone [10,11]. Furthermore, the AIP is known to be correlated with sleep disorders and the apnea-hypopnea index in previous studies. However, most of these studies have been small-scale cross-sectional studies [12], and none have been conducted on the general population in Korea.

This study aimed to evaluate OSA risk using the STOP-BANG scoring system and to explore its correlation with atherosclerosis risk, which is calculated by AIP. Through this research, we hope to highlight the broader atherosclerotic and metabolic implications of OSA and reinforce the importance of effective screening and management strategies.

Methods

Study participants

This is a cross-sectional study based on data from the Korea National Health and Nutrition Examination Survey (KNHANES) conducted between 2019 and 2021. The survey included 8,110 participants in 2019, 7,359 participants in 2020, and 7,090 participants in 2021, resulting in a total sample size of 22,559 participants. This trial

was approved by institutional review board of Yeungnam University Hospital (2024-07-034). In addition, we utilized fully anonymized data from the KNHANES. The study authors did not have access to any information that could identify individual participants during or after study. For this reason, the requirement for informed consent was waived by the IRB of Yeungnam University Hospital.

Participants with a measurable STOP-BANG score were included in the study, which accounted for 11,423 individuals. Exclusion criteria, which were applied to ensure the reliability and relevance of the data, were participants who were unable to calculate the AIP (n = 242), had a history of cerebrovascular accident (n = 342), had a history of myocardial infarction (n = 179), had aspartate aminotransferase (AST) levels ≥200 or alanine aminotransferase (ALT) levels ≥175 (n = 18), had liver cirrhosis (n = 52), had current cancer (n = 290), had a glomerular filtration rate ≤15 mL/min/1.73 m^2 (n = 10), or were currently taking medication for dyslipidemia (n = 2,573). The current study investigated the correlation between the STOP-BANG score and the AIP. To ensure the accuracy of the results, we excluded conditions that could affect cholesterol levels, such as severe liver disease, which affects lipid profile due to alteration in hepatic synthesis of cholesterol [13,14]; end-stage renal disease, which alters cholesterol metabolism and causes impaired high-density lipoprotein cholesterol (HDL-C) function [15,16]; additionally, cancer and its treatment can significantly alter patient's physiology, including their immune system, lipid metabolism, and overall health [17]. After these exclusions, 8,158 participants were included in the final analysis (Fig. 1).

Fig. 1. Patient flowchart

Clinical and laboratory measurements

Data collection involved demographic information such as age and sex, clinical measurements including BMI and blood pressure, and laboratory tests such as lipid profiles and liver enzymes. Lifestyle factors such as current smoking, high-risk alcohol consumption, aerobic exercise, and resistance exercise, as well as socioeconomic factors such as education and household income, were included. Current smokers are defined as people who have smoked more than five packs (100 cigarettes) in their lifetime and are currently smoking. High-risk alcohol consumption is defined as people who drink, on average, more than seven glasses at one time for men and more than five glasses for women and drink more than two times a week. Aerobic exercise is defined as engaging in at least 2 h and 30 min of moderate-intensity physical activity per week, at least 1 h and 15 min of vigorous-intensity physical activity per week, or a combination of moderate and vigorous-intensity activities (with 1 min of vigorous-intensity activity being equivalent to 2 min of moderate-intensity activity) to meet the recommended amount of physical activity. Resistance exercise is defined as individuals who have engaged in resistance exercise exercises such as push-ups, sit-ups, dumbbells, barbells, or pull-ups for at least 2 d in the past week. The education variable was categorized into four groups according to the highest level of education attained: elementary school or lower, middle school graduate, high school graduate, college graduate, or higher. The household income was defined by household income quartiles.

The laboratory tests included fasting glucose (mg/dL), glycated hemoglobin (HbA1c) (%), total cholesterol (mg/dL), HDL-C (mg/dL), triglycerides (TG, mg/dL), and calculated LDL-C (total cholesterol – [TG/5 + HDL-C]), which was used instead of direct LDL-C because of many missing values. AST (IU/mL), ALT (IU/mL), blood urea

nitrogen (mg/dL), and creatine (mg/dL) were included. The presence of diabetes in patients was classified based on HbA1c levels, fasting glucose levels, use of diabetes medication, and physician diagnosis. Normal is defined as having a fasting glucose level <100 mg/dL or an HbA1c ≤5.7%. Prediabetes is defined as having a fasting glucose level between 100 and 125 mg/dL or an HbA1c between 5.7% and 6.4%. Diabetes is defined as having a fasting glucose level ≥126 mg/dL, taking oral hypoglycemic agents, receiving insulin injections, having a previous doctor's diagnosis, or having an HbA1c ≥6.5% [18]. The homeostatic model assessment for insulin resistance (HOMA-IR) and Homeostatic Model Assessment for β-cell function (HOMA-β) were calculated using the following formulas:

HOMA-IR = fasting insulin (μU/mL) × fasting glucose (mg/dL)/405

and

HOMA-β = 20 × fasting insulin (μU/mL)/fasting glucose (mg/dL)–3.5.

Assessment of OSA

The STOP-BANG score, a validated screening tool for OSA, comprises eight questions that assess risk factors related to OSA. Each item is scored as either 0 or 1, with a total possible score ranging from 0 to 8 [8]. The components of the STOP-BANG questionnaire are the following:

S: snoring loudly (louder than talking or loud enough to be heard through closed doors)

T: tiredness, i.e., feeling tired, fatigued, or sleepy during the daytime

O: observed apnea, i.e., observed patients stop breathing while sleeping

P: high blood pressure

B: BMI >35 kg/m^2 [8]. However, a lower BMI threshold should be set for the Asian population. In a study involving 1,205 individuals of different ethnicities, the optimal area under the receiver operating characteristic curve value for BMI to diagnose high-risk OSA was lower for Chinese and Indian individuals [19]. In this study, we set the threshold at 30 kg/m^2.

A: age >50 years

N: neck circumference >40 cm

G: gender (1 = men; 0 = women) [8].

Participants were classified based on their STOP-BANG scores into three risk OSA categories: low risk (0–2 points), intermediate risk (3–4 points), and high risk (≥5 points). Additionally, participants in the intermediate-risk category were reclassified as high-risk if they scored ≥2 on the STOP portion and were male, had a BMI ≥30, or had a neck circumference >40 cm [8].

The reliability of the STOP-BANG score has been published elsewhere. With a stepwise increase in the STOP-BANG score from 3 to 8, the probability of severe OSA increases from 15% to 75% in the general population [20], and a score >5 is reliable for detecting severe OSA with 90% to 100% sensitivity [8,21,22]. The STOP-BANG questionnaire effectively screens for OSA, with a sensitivity of up to 93.9% and an area under the curve of 0.86 in patients with cardiovascular risk factors [23].

Assessment of atherogenic risk

We calculated the AIP for the assessment of atherosclerosis and metabolic dysfunction. The AIP is a logarithmic transformation of the ratio of TGs to HDL-C (log[TG/HDL-C]), and an AIP value >0.24 is considered high risk for cardiovascular events [12,24,25].

Statistical analyses

We analyzed categorical variables using a chi-square test and expressed the data as percentages. Continuous variables were analyzed with an analysis of variance to compare across the groups, and the results were shown as means with a standard deviation. Tukey's HSD post-hoc analysis was conducted to compare differences between the groups.

To evaluate the atherogenic risk associated with low, intermediate, and high STOP-BANG scores, a logistic regression analysis was conducted. This approach allowed us to calculate the odds ratios (ORs) for high AIP with high and intermediate STOP-BANG scores relative to those with low STOP-BANG scores. Covariates such as sex, age, smoking status, alcohol consumption, education, income, aerobic exercise, resistance exercise, diabetes mellitus, HbA1c, and HOMA-IR were included in the logistic regression models to control for potential confounding factors. Model 1 was adjusted for sex and age. Model 2 was adjusted for model 1 + smoking, high-risk alcohol consumption, education, house income, aerobic exercise, and resistance exercise. Model 3 was adjusted for model 2 + diabetes, HbA1c, and HOMA-IR. The results of these analyses provided adjusted odds ratios, indicating the strength of the association between high STOP-BANG scores and elevated metabolic risks, independent of these confounders. Statistical significance was assessed with two-tailed tests and has a cutoff p-value <0.05. All statistical analyses were performed using R for Windows, version 4.4

(The R Project, Vienna, Austria).

Results

Baseline characteristics

Table 1 provides the clinical characteristics of the study population, which includes 8,158 participants (male, n = 3,389, 41.5%; female, n = 4,769, 58.5%; average age, 57.9 years). Participants were categorized by OSA risk based on the STOP-BANG questionnaire. Specifically, 4,681 participants (57.4%) were classified as having low risk, 2,421 (29.7%) as having intermediate risk, and 1,056 (12.9%) as having high risk. The high-OSA risk group had a higher proportion of male participants, elevated blood pressure, and a higher BMI than the low- and intermediate-OSA risk groups. Additionally, the high-OSA risk group had higher rates of current smokers and high-risk alcohol drinkers. This group had a higher prevalence of diabetes and elevated fasting glucose and HOMA-IR levels. In terms of lipid profiles, the high-risk group had higher TG levels and lower HDL-C levels.

The AIP values were –0.09 ± 0.30, 0.06 ± 0.31, and 0.13 ± 0.32 for the low-, intermediate-, and high-OSA risk groups, respectively ($p < 0.001$). Figure 2 shows the proportion of atherogenic risk (AIP >0.24) according to the STOP-BANG score. In the high-OSA risk group, 34.7% were at atherogenic risk, which was significantly higher than that of the intermediate- and low-risk groups (27.6% and 13.7%, respectively; $p < 0.001$).

Table 1. Clinical characteristics

	Total	STOP BANG score			P-value
		Low	Intermediate	High	
	(N=8,158)	(N=4,681)	(N=2,421)	(N=1,056)	
Age(yr)	57.9 ± 11.7	55.7 ± 11.6	61.8 ± 11.0	58.2 ± 11.4	<0.001
Sex					<0.001
Male	3389(41.5)	729 (15.6)	1716 (70.9)	944 (89.4)	
Female	4769(58.5)	3952 (84.4)	705 (29.1)	112 (10.6)	
SBP(mmHg)	121.1 ± 16.7	117.0 ± 15.7	126.1 ± 16.7	127.6 ± 15.6	<0.001
DBP(mmHg)	76.0 ± 9.9	74.1 ± 8.9	77.6 ± 9.9	81.1 ±11.1	<0.001
BMI(kg/m^2)	23.8 ± 3.4	23.0 ± 3.0	24.6 ± 3.2	26.1 ± 3.9	<0.001
Current Smoker	1248 (15.3)	415(8.9)	542(22.4)	291(27.6)	<0.001
High risk Alcohol consumption	846 (10.4)	278(5.9)	334(13.8)	234(22.2)	<0.001
Aerobic exercise	3190 (39.1)	1849 (39.5)	944 (39.0)	397 (37.6)	0.5139
Resistance training	1699 (20.8)	853 (18.2)	597 (24.7)	249 (23.6)	<0.001
House Income					<0.001
1quartile	1566 (19.3)	779 (16.7)	587 (24.3)	200 (19.0)	
2quartile	1991 (24.5)	1143 (24.5)	576 (23.9)	272 (25.8)	
3quartile	2159 (26.6)	1280 (27.5)	615 (25.5)	264 (25.0)	
4quartile	2406 (29.6)	1455 (31.2)	633 (26.3)	318 (30.2)	
Education					<0.001
Elemental school or lower	1676 (20.6)	870 (18.6)	600 (24.8)	206 (19.5)	
Middle school	909 (11.1)	469 (10.0)	322 (13.3)	118 (11.2)	

High school	2738 (33.6)	1611 (34.4)	788 (32.6)	339 (32.1)	
College graduate or higher	2830 (34.7)	1727 (36.9)	710 (29.3)	393 (37.2)	
Prediabetes	3914 (49.1)	2143(46.7)	1234(52.4)	537(52.1)	<0.001
Diabetes	1078(13.5)	387(8.4)	445(18.9)	246(23.9)	<0.001
Fasting Glucose(mg/dl)	102.5±22.6	98.9±19.8	106.6±24.8	108.7±25.7	<0.001
HbA1c(%)	5.8±0.8	5.7±0.7	6.0±0.9	6.0±0.9	<0.001
HOMA-IR	2.2±2.4	1.9±1.7	2.5±2.9	3.0±3.1	<0.001
HOMA-β	81.4±74.3	79.0±78.8	81.2±63.0	92.7±77.2	<0.001
Total Cholesterol(mg/dl)	200.7±38.0	203.8±36.7	196.1±38.4	197.7±40.8	<0.001
HDL-Cholesterol(mg/dl)	52.1±12.9	55.1±13.0	48.6±11.7	46.8±11.2	<0.001
Triglyceride(mg/dl)	133.8±109.3	116.0±80.0	150.2±124.2	175.1±157.8	<0.001
LDL-Cholesterol(mg/dl)	121.9±35.6	125.4±33.5	117.6±36.5	116.1±40.3	<0.001
AST(IU/ml)	24.6±10.9	23.2±9.0	26.2±12.6	27.5±13.1	<0.001
ALT(IU/ml)	22.1±14.5	19.3±11.8	24.6±15.5	29.0±19.3	<0.001
BUN(mg/dl)	15.5±4.6	14.8±4.4	16.4±4.7	16.4±4.9	<0.001
CRE(mg/dl)	0.8±0.2	0.7±0.1	0.9±0.2	0.9±0.2	<0.001
eGFR(mL/min/1.73m^2)	95.9±13.9	98.6±12.9	92.0±14.0	92.7±15.2	<0.001
AIP	-0.02±0.31	-0.09±0.3	0.06±0.31	0.13±0.32	<0.001

OSA, obstructive sleep apnea; SBP, systolic blood pressure; DBP, diastolic blood pressure; BMI, body mass index; HOMA-IR, Homeostatic Model Assessment for Insulin Resistance; HOMA- β, homeostatic model assessment of β-cell function; HDL, high-density lipoprotein; LDL, low-density lipoprotein; AST, aspartate aminotransferase; ALT, alanine aminotransferase; BUN, blood urea nitrogen; CRE, creatinine; eGFR, estimated glomerular filtration rate; AIP, atherogenic index of plasma; AIP calculated by logarithm[Triglyceride/HDL-C]

Fig. 2. Proportion of atherogenic risk (AIP >0.24) according to OSA risk

Comparison of intermediate or high OSA risk to low OSA risk: ***p <0.001. AIP, atherogenic index of plasma; OSA, obstructive sleep apnea

Effect of OSA on atherogenic risk

The effect of OSA on atherogenic risk (AIP >0.24) was explored using logistic regression analysis (Table 2). An unadjusted model showed that, compared with the low-OSA risk group, the high-OSA risk group had a significantly greater risk of having AIP >0.24 (OR, 3.34; 95% CI, 2.86–3.88; p <0.001). The intermediate-OSA risk group showed a significantly greater risk than the low-OSA risk group (OR, 2.40; 95% CI, 2.12–2.71; p <0.001). Even after adjusting for confounding variables such as sex, age, sociological factors (household income, education), lifestyle factors (smoking, alcohol consumption, exercise), and comorbidities such as diabetes, the high-OSA risk group still showed a significant association with AIP >0.24 (OR, 1.42; 95% CI, 1.16–1.73; p <0.001), as did the intermediate-OSA risk group (OR, 1.53; 95% CI, 1.30–1.79; p <0.001). The presence of diabetes was significantly associated with an increased risk of AIP >0.24; compared with normoglycemic participants, the ORs for AIP >0.24 were 1.69 (95% CI, 1.46–1.96) and 1.71 (95% CI, 1.32–2.23) for those with prediabetes and diabetes, respectively (Table 3).

Table 2. Effect of OSA on atherogenic risk (AIP > 0.24)

	Crude		Model 1		Model 2		Model 3	
	OR(95% CI)	p	OR(95% CI)	p	OR(95% CI)	p	OR(95% CI)	p
OSA risk								
Low	1(ref)		1 (ref)		1 (ref)		1 (ref)	
Intermediate	2.40 (2.12-2.71)	<0.001	1.87 (1.60-2.17)	<0.001	1.83 (1.58-2.13)	<0.001	1.35 (1.16-1.58)	<0.001

High	3.34 (2.86-3.88)	<0.001	2.28 (1.89-2.73)	<0.001	2.17 (1.81-2.61)	<0.001	1.32 (1.08-1.61)	0.006

Logistic regression analysis was performed.

Crude: Unadjust model. Model1: adjusted for sex and age. Model 2: adjusted for model1 + current smoking, high risk alcohol consumption, education, house income, aerobic exercise and resistance training. Model 3: adjusted for model2 + diabetes, HbA1c, HOMA-IR.

Table 3. Multivariate logistic regression analysis of presence of diabetes for high AIP(>0.24)

	Crude		Model 1		Model 2		Model 3	
	OR(95% CI)	P-value	OR(95% CI)	P-value	OR(95% CI)	P-value	OR(95% CI)	P-value
normal	1(ref)		1 (ref)		1 (ref)		1 (ref)	
Prediabetes	2.19 (2.05-2.71)	<0.001	2.26 (1.97-2.59)	<0.001	2.28 (1.98-2.62)	<0.001	1.67 (1.44-1.93)	<0.001
Diabetes	3.64 (2.75-3.91)	<0.001	3.70 (3.10-4.43)	<0.001	3.57 (2.98-4.28)	<0.001	1.49 (1.14-1.94)	0.003

Crude: Unadjust model. Model1: adjusted for sex and age. Model 2: adjusted for model1 + current smoking, high risk alcohol consumption, education, house income, aerobic exercise and resistance training. Model 3: adjusted for model2 + HbA1c, HOMA-IR.

Effect of OSA on atherogenic risk according to the presence of diabetes

Patients were classified as non-diabetic (normal and prediabetes) and diabetic, and the association between atherogenic risk according to OSA risk groups was further analyzed. We examined fasting glucose, HbA1c, HOMA-IR, and AIP among participants with and without diabetes according to OSA risk, and the results are represented in Fig. 3&4. The levels of fasting glucose and HbA1c did not differ according to OSA risks regardless of the presence of diabetes (Fig. 3A, 3B and 4A, 4B). However, HOMA-IR and AIP levels and the proportion of atherogenic risk had an increasing tendency with statistical significance according to OSA risks among participants with and without diabetes (Fig.

3C, 3D, 3E and 4C, 4D, 4E).

Fig. 3. Comparison of metabolic parameters and AIP based on OSA risk in populations without diabetes

Fig. 4. Comparison of metabolic parameters and AIP based on OSA risk in populations with diabetes

Comparison of intermediate or high OSA risk to low OSA risk: *p <0.05, **p <0.01, ***p <0.001. AIP, atherogenic index of plasma; OSA, obstructive sleep apnea

The effect of OSA on atherogenic risk among participants with and without diabetes is discussed in Table 4. Logistic regression analysis models were adjusted for covariates considered in Table 2, except for the presence of diabetes. After multivariate adjustments, among participants without diabetes, intermediate (OR, 1.21; 95% CI, 1.02–1.45) but not high OSA risk (OR, 1.17; 95% CI, 0.93–1.47) was an independent factor for atherogenic risk. Meanwhile, among participants with diabetes, intermediate (OR, 1.51; 95% CI, 1.05–2.19) and high OSA risk (OR, 1.58; 95% CI, 1.02–2.46) significantly increased the risk of atherogenic risk.

Table 4. Effect of OSA on atherogenic risk (AIP >0.24) according to the presence of diabetes

**(A) Normal + Prediabetes**	Crude		Model 1		Model 2		Model 3	
	OR(95% CI)	P	OR(95% CI)	P	OR(95% CI)	P	OR(95% CI)	P

OSA risk								
Low	1(ref)		1 (ref)		1 (ref)		1 (ref)	
Intermediate	2.36 (2.06-2.71)	<0.001	1.76 (1.48-2.08)	<0.001	1.73 (1.46-2.06)	<0.001	1.21 (1.02-1.45)	0.03
High	3.28 (2.75-3.91)	<0.001	2.10 (1.71-2.59)	<0.001	2.04 (1.65-2.52)	<0.001	1.17 (0.93-1.47)	0.17
**(B) Diabetes**	Crude		Model 1		Model 2		Model 3	
	OR(95% CI)	P	OR(95% CI)	P	OR(95% CI)	P	OR(95% CI)	P
OSA risk								
Low	1(ref)		1 (ref)		1 (ref)		1 (ref)	
Intermediate	1.72 (1.27-2.32)	<0.001	1.65 (1.15-2.36)	<0.001	1.62 (1.13-2.32)	0.009	1.51 (1.05-2.19)	0.03
High	2.22 (1.58-3.13)	<0.001	1.82 (1.20-2.76)	<0.001	1.74 (1.14-2.66)	0.01	1.58 (1.02-2.46)	0.04

Crude: Unadjust model. Model1: adjusted for sex and age. Model 2: adjusted for model1 + current smoking, high risk alcohol consumption, education, house income, aerobic exercise and resistance training. Model 3: adjusted for model2 + HbA1c, HOMA-IR.

Discussion

In this study, we found the close association between higher OSA risk and atheroscelrogenic risk in the Korean population from nationwide cross-sectional study based on KNHANES data. Intermediate or high OSA risk assessed by the STOP-BANG score was associated with a 1.3-fold increased risk of atherogenesis (AIP>0.24), and these associations remained robust even after adjusting for multiple confounding variables, particularly among those with diabetes.

The OSA risk calculated using the STOP-BANG questionnaire is associated with

diabetes and metabolic syndrome. Previously, two studies that used KNHANES 2019–2020 reported that patients with diabetes mellitus, hypertension, and obesity have a higher risk of OSA, which increased synergistically when they concurrently had two or more of these conditions [26] and that a high STOP-BANG score is related to a higher risk of insulin resistance and metabolic syndrome [27]. Despite this, OSA risk is often inadequately assessed in clinical practice. The STOP-BANG questionnaire is simple and can be quickly completed by patients without specialized knowledge. Its ease of use makes it a practical tool for routine screening, highlighting its clinical usefulness in improving early detection and management of OSA, thereby enhancing the management of patients' metabolic risks.

AIP is used to assess the risk of atherosclerosis and CVDs. AIP is easily obtainable and based on lipid profiles, including TG and HDL-C. Although the definite mechanism of the relationship between OSA and lipid profile has not been established, it is suggested that chronic intermittent hypoxia may elevate TG levels by enhancing enzymes and proteins involved in TG production in the liver [28]. Furthermore, chronic intermittent hypoxemia is regarded as the primary mechanism that contributes to IR in OSA [29]. Previous studies have reported that the AIP value increases with increasing cardiovascular risk and is a superior predictor of CVD than individual lipid risk factors [11,24,30]. Another cross-sectional study involving 560 patients reported that OSA severity was independently associated with AIP, regardless of obesity, and that AIP demonstrated superior predictive value for nocturnal hypoxemia than individual lipid risk factors [31].

We investigated the relationship between the risk of OSA and AIP with respect to the presence of diabetes, as diabetes is a significant variable that increases the risk of

atherosclerosis. Furthermore, a short sleep duration increases the risk of developing type 2 diabetes [32]. OSA contributes to diabetes through intermittent hypoxia, increased sympathetic activity, and elevated inflammation, which impair glucose homeostasis and insulin sensitivity [33]. In a previous study that included patients with type 2 diabetes, a STOP-BANG score ≥5 was associated with increased insulin resistance and was a predictor of 10-year fatal and non-fatal coronary heart disease risk [34]. In another cross-sectional study, a high risk of OSA (STOP-BANG score ≥5) showed a positive correlation with mean HbA1c and fasting glucose levels in patients with diabetes [35]. In our study, there was no significant difference in HbA1c and fasting glucose according to the STOP-BANG score among patients with diabetes. However, we observed a significant increase in HOMA-IR and AIP as the risk of OSA increased. These findings suggest that a high risk of OSA does lead to a sleep disorder, a complex condition with profound implications for cardiovascular health. Even among patients with diabetes with similar blood glucose levels, an increased OSA risk is associated with higher cardiometabolic dysfunction. Therefore, the STOP-BANG may be used as a simple predictor of atherogenic risk in diabetes care settings.

This study has several limitations. First, the absence of polysomnography results, which are the gold standard for diagnosing OSA, limits the precision of our findings. The STOP-BANG score is known for its low specificity despite having a high sensitivity of up to 92% to 100% for severe OSA [8]. This means that the STOP-BANG score potentially inflates the associations observed between OSA and metabolic disorders. However, polysomnography is not feasible for large-scale studies of the general population owing to its high cost and limited accessibility [7,8]. In previous studies based on polysomnography, the sample sizes were relatively small, and

participants were likely individuals already aware of their symptoms [25,31,36]. This suggests the possibility of selection bias, with more severe cases of OSA being overrepresented. Second, because this is a cross-sectional study, our research has limited ability to establish causality. Longitudinal studies with follow-up data are needed to better understand the directionality and causative pathways underlying these associations.

Despite these limitations, the strength of the current study is that it uses the STOP-BANG score to assess a large sample of the Korean general population. Our study highlights important associations between high STOP-BANG scores and atherogenic risk, underscoring the need for further research and integrated clinical approaches to address both sleep health and metabolic conditions.

The implications of these findings are significant. Our research highlights the importance of considering OSA, in the context of atherogenic risk. Clinicians should be aware that patients with high STOP-BANG scores may be at increased risk for atherogenic disturbances and should consider early interventions to manage these risks, potentially reducing the risk of CVD and other related complications.

In conclusion, OSA is linked to an increased atherogenic risk, particularly in patients with diabetes. Screening for high-risk OSA using the STOP-BANG might be beneficial in the diabetes care setting to prevent cardiometabolic complications. Further prospective studies that could provide deeper insights into the underlying mechanisms and potential benefits of OSA treatment in reducing atherogenic risk are warranted.

Acknowledgements

Not applicable

References

1. Benjafield AV, Ayas NT, Eastwood PR, Heinzer R, Ip MSM, Morrell MJ, et al. Estimation of the global prevalence and burden of obstructive sleep apnoea: a literature-based analysis. Lancet Respir Med. 2019; 7:687-698. https://doi.org/10.1016/s2213-2600(19)30198-5 PMID: 31300334
2. Togeiro SM, Carneiro G, Ribeiro Filho FF, Zanella MT, Santos-Silva R, Taddei JA, et al. Consequences of obstructive sleep apnea on metabolic profile: a Population-Based Survey. Obesity (Silver Spring). 2013; 21:847-851. https://doi.org/10.1002/oby.20288 PMID: 23712988
3. Amin Z, Amin HZ, Amin LZ. Obstructive Sleep Apnea and Atherosclerosis. Acta Med Indones. 2016; 48:63-67 PMID: 27241547
4. Pafili K, Steiropoulos P, Papanas N. The relationship between obstructive sleep apnoea and coronary heart disease. Curr Opin Cardiol. 2015; 30:439-446. https://doi.org/10.1097/hco.0000000000000172 PMID: 26049394
5. Xie C, Zhu R, Tian Y, Wang K. Association of obstructive sleep apnoea with the risk of vascular outcomes and all-cause mortality: a meta-analysis. BMJ Open. 2017; 7:e013983. https://doi.org/10.1136/bmjopen-2016-013983 PMID: 29275335
6. Kapur V, Strohl KP, Redline S, Iber C, O'Connor G, Nieto J. Underdiagnosis of sleep apnea syndrome in U.S. communities. Sleep Breath. 2002; 6:49-54. https://doi.org/10.1007/s11325-002-0049-5 PMID: 12075479
7. Chung F, Abdullah HR, Liao P. STOP-Bang Questionnaire: A Practical Approach to Screen for Obstructive Sleep Apnea. Chest. 2016; 149:631-638. https://doi.org/10.1378/chest.15-0903 PMID: 26378880
8. Chung F, Yegneswaran B, Liao P, Chung SA, Vairavanathan S, Islam S, et al. STOP questionnaire: a tool to screen patients for obstructive sleep apnea. Anesthesiology. 2008; 108:812-821. https://doi.org/10.1097/ALN.0b013e31816d83e4 PMID: 18431116
9. Valdivia G, Schmidt A, Schmidt B, Rivera F, Oñate A, Navarrete C, et al. Association between cardiovascular mortality and STOP-Bang questionnaire scores in a cohort of hospitalized patients: a prospective study. J Bras Pneumol. 2021; 47:e20210039. https://doi.org/10.36416/1806-3756/e20210039 PMID: 34495174
10. Millán J, Pintó X, Muñoz A, Zúñiga M, Rubiés-Prat J, Pallardo LF, et al.

Lipoprotein ratios: Physiological significance and clinical usefulness in cardiovascular prevention. Vasc Health Risk Manag. 2009; 5:757-765 PMID: 19774217

11. Cai G, Shi G, Xue S, Lu W. The atherogenic index of plasma is a strong and independent predictor for coronary artery disease in the Chinese Han population. Medicine (Baltimore). 2017; 96:e8058. https://doi.org/10.1097/md.0000000000008058 PMID: 28906400

12. Cao B, Fan Z, Zhang Y, Li T. Independent association of severity of obstructive sleep apnea with lipid metabolism of atherogenic index of plasma (AIP) and apoB/apoAI ratio. Sleep Breath. 2020; 24:1507-1513. https://doi.org/10.1007/s11325-020-02016-1 PMID: 31981042

13. Privitera G, Spadaro L, Marchisello S, Fede G, Purrello F. Abnormalities of Lipoprotein Levels in Liver Cirrhosis: Clinical Relevance. Dig Dis Sci. 2018; 63:16-26. https://doi.org/10.1007/s10620-017-4862-x PMID: 29177578

14. Sabesin SM. Lipid and lipoprotein abnormalities in alcoholic liver disease. Circulation. 1981; 64:Iii 72-84 PMID: 7020988

15. Vaziri ND. Causes of dysregulation of lipid metabolism in chronic renal failure. Semin Dial. 2009; 22:644-651. https://doi.org/10.1111/j.1525-139X.2009.00661.x PMID: 20017835

16. Trevisan R, Dodesini AR, Lepore G. Lipids and renal disease. J Am Soc Nephrol. 2006; 17:S145-147. https://doi.org/10.1681/asn.2005121320 PMID: 16565240

17. Bian X, Liu R, Meng Y, Xing D, Xu D, Lu Z. Lipid metabolism and cancer. J Exp Med. 2021; 218. https://doi.org/10.1084/jem.20201606 PMID: 33601415

18. Korea Disease Control and Prevention Agency. The Eighth Korea National Health and Nutrition Examination Survey (KNHANES Ⅷ-3). 2021

19. Waseem R, Chan MTV, Wang CY, Seet E, Tam S, Loo SY, et al. Diagnostic performance of the STOP-Bang questionnaire as a screening tool for obstructive sleep apnea in different ethnic groups. J Clin Sleep Med. 2021; 17:521-532. https://doi.org/10.5664/jcsm.8940 PMID: 33112227

20. Nagappa M, Liao P, Wong J, Auckley D, Ramachandran SK, Memtsoudis S, et al. Validation of the STOP-Bang Questionnaire as a Screening Tool for Obstructive Sleep Apnea among Different Populations: A Systematic Review and Meta-Analysis. PLoS One. 2015; 10:e0143697. https://doi.org/10.1371/journal.pone.0143697 PMID: 26658438

21. Oshita H, Ito N, Senoo M, Funaishi K, Mitama Y, Okusaki K. The STOP-Bang Test Is Useful for Predicting the Severity of Obstructive Sleep Apnea. Jma j. 2020; 3:347-352. https://doi.org/10.31662/jmaj.2020-0002 PMID: 33225107
22. Chiu HY, Chen PY, Chuang LP, Chen NH, Tu YK, Hsieh YJ, et al. Diagnostic accuracy of the Berlin questionnaire, STOP-BANG, STOP, and Epworth sleepiness scale in detecting obstructive sleep apnea: A bivariate meta-analysis. Sleep Med Rev. 2017; 36:57-70. https://doi.org/10.1016/j.smrv.2016.10.004 PMID: 27919588
23. Hwang M, Zhang K, Nagappa M, Saripella A, Englesakis M, Chung F. Validation of the STOP-Bang questionnaire as a screening tool for obstructive sleep apnoea in patients with cardiovascular risk factors: a systematic review and meta-analysis. BMJ Open Respir Res. 2021; 8. https://doi.org/10.1136/bmjresp-2020-000848 PMID: 33664122
24. Dobiásová M. [AIP--atherogenic index of plasma as a significant predictor of cardiovascular risk: from research to practice]. Vnitr Lek. 2006; 52:64-71 PMID: 16526201
25. Bikov A, Meszaros M, Kunos L, Negru AG, Frent SM, Mihaicuta S. Atherogenic Index of Plasma in Obstructive Sleep Apnoea. J Clin Med. 2021; 10. https://doi.org/10.3390/jcm10030417 PMID: 33499142
26. Huh G, Han KD, Park YM, Park CS, Lee KN, Lee EY, et al. Comorbidities associated with high-risk obstructive sleep apnea based on the STOP-BANG questionnaire: a nationwide population-based study. Korean J Intern Med. 2022; 38:80-92. https://doi.org/10.3904/kjim.2022.198 PMID: 36353789
27. Kim T, Kang J. Relationship between obstructive sleep apnea, insulin resistance, and metabolic syndrome: a nationwide population-based survey. Endocr J. 2023; 70:107-119. https://doi.org/10.1507/endocrj.EJ22-0280 PMID: 36171092
28. Adedayo AM, Olafiranye O, Smith D, Hill A, Zizi F, Brown C, et al. Obstructive sleep apnea and dyslipidemia: evidence and underlying mechanism. Sleep Breath. 2014; 18:13-18. https://doi.org/10.1007/s11325-012-0760-9 PMID: 22903801
29. Gabryelska A, Karuga FF, Szmyd B, Białasiewicz P. HIF-1α as a Mediator of Insulin Resistance, T2DM, and Its Complications: Potential Links With Obstructive Sleep Apnea. Front Physiol. 2020; 11:1035. https://doi.org/10.3389/fphys.2020.01035 PMID: 33013447

30. Lioy B, Webb RJ, Amirabdollahian F. The Association between the Atherogenic Index of Plasma and Cardiometabolic Risk Factors: A Review. Healthcare (Basel). 2023; 11. https://doi.org/10.3390/healthcare11070966 PMID: 37046893
31. Bikov A, Frent SM, Meszaros M, Kunos L, Mathioudakis AG, Negru AG, et al. Triglyceride-Glucose Index in Non-Diabetic, Non-Obese Patients with Obstructive Sleep Apnoea. J Clin Med. 2021; 10. https://doi.org/10.3390/jcm10091932 PMID: 33947164
32. Lee DY, Jung I, Park SY, Yu JH, Seo JA, Kim KJ, et al. Sleep Duration and the Risk of Type 2 Diabetes: A Community-Based Cohort Study with a 16-Year Follow-up. Endocrinol Metab (Seoul). 2023; 38:146-155. https://doi.org/10.3803/EnM.2022.1582 PMID: 36740966
33. Song SO, He K, Narla RR, Kang HG, Ryu HU, Boyko EJ. Metabolic Consequences of Obstructive Sleep Apnea Especially Pertaining to Diabetes Mellitus and Insulin Sensitivity. Diabetes Metab J. 2019; 43:144-155. https://doi.org/10.4093/dmj.2018.0256 PMID: 30993938
34. Protasiewicz Timofticiuc DC, Vladu IM, Ștefan AG, Clenciu D, Mitrea A, Pădureanu V, et al. Associations of Chronic Diabetes Complications and Cardiovascular Risk with the Risk of Obstructive Sleep Apnea in Patients with Type 2 Diabetes. J Clin Med. 2022; 11. https://doi.org/10.3390/jcm11154403 PMID: 35956020
35. Mehrdad M, Azarian M, Sharafkhaneh A, Alavi A, Leili EK, Rad AH, et al. The association between OSA and glycemic control in diabetes. Int J Prev Med. 2023; 14:26. https://doi.org/10.4103/ijpvm.ijpvm_356_21 PMID: 37033275
36. Kang HH, Kim SW, Lee SH. Association between triglyceride glucose index and obstructive sleep apnea risk in Korean adults: a cross-sectional cohort study. Lipids Health Dis. 2020; 19:182. https://doi.org/10.1186/s12944-020-01358-9 PMID: 32771021

Figure1

Click here to access/download;Figure;FIG1.tiff

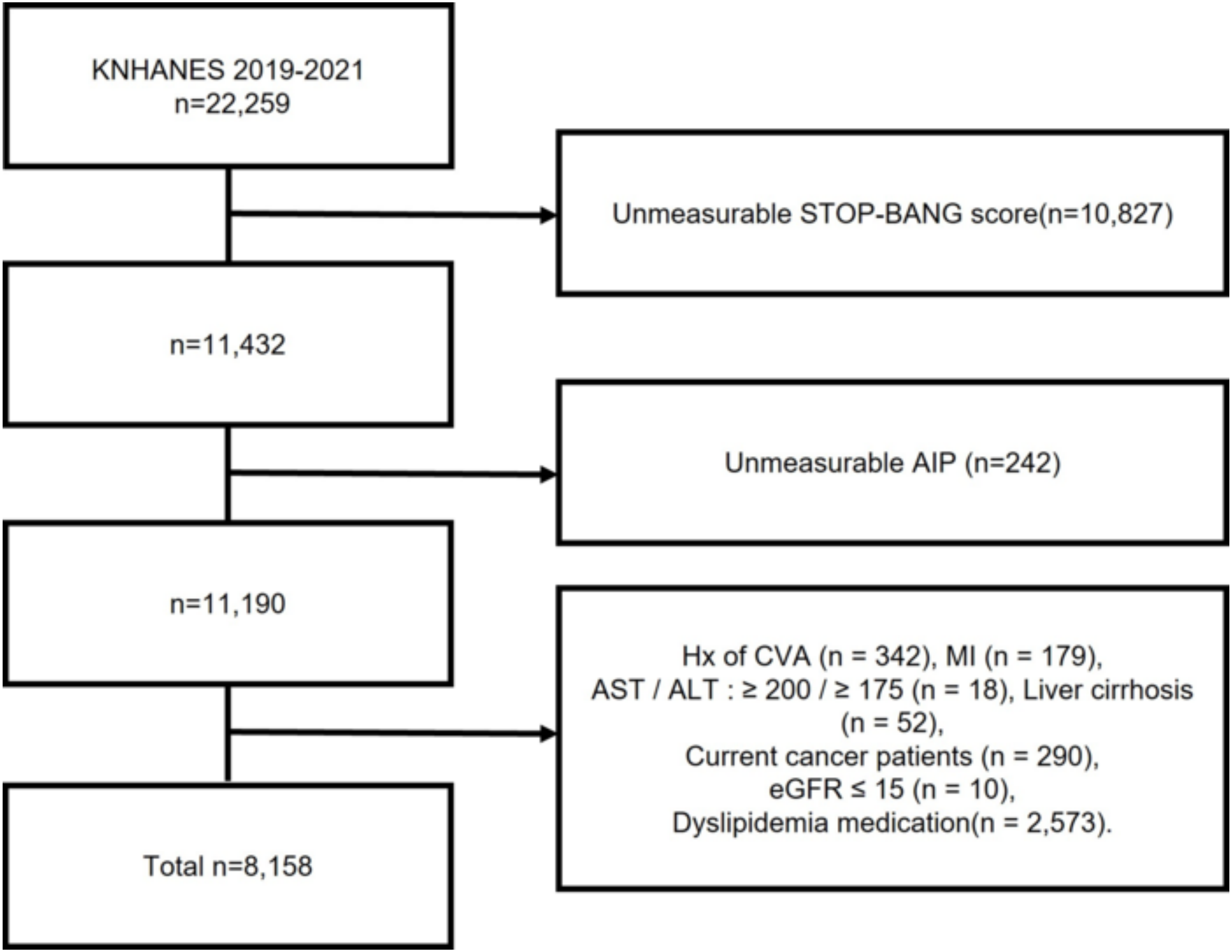

Figure2

Click here to access/download;Figure;FIG2.tiff

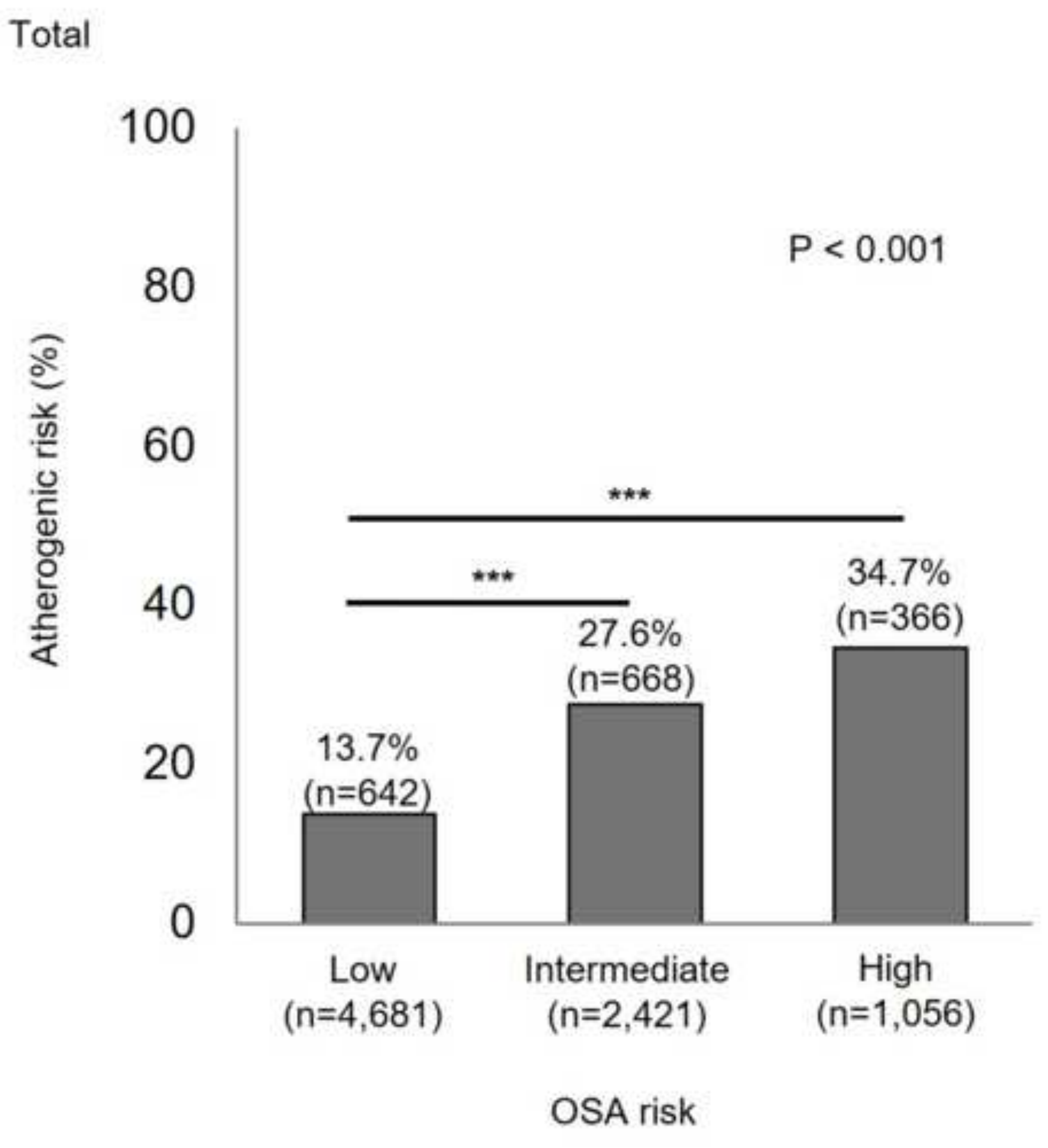

Figure3

Click here to access/download;Figure;FIG3.tiff

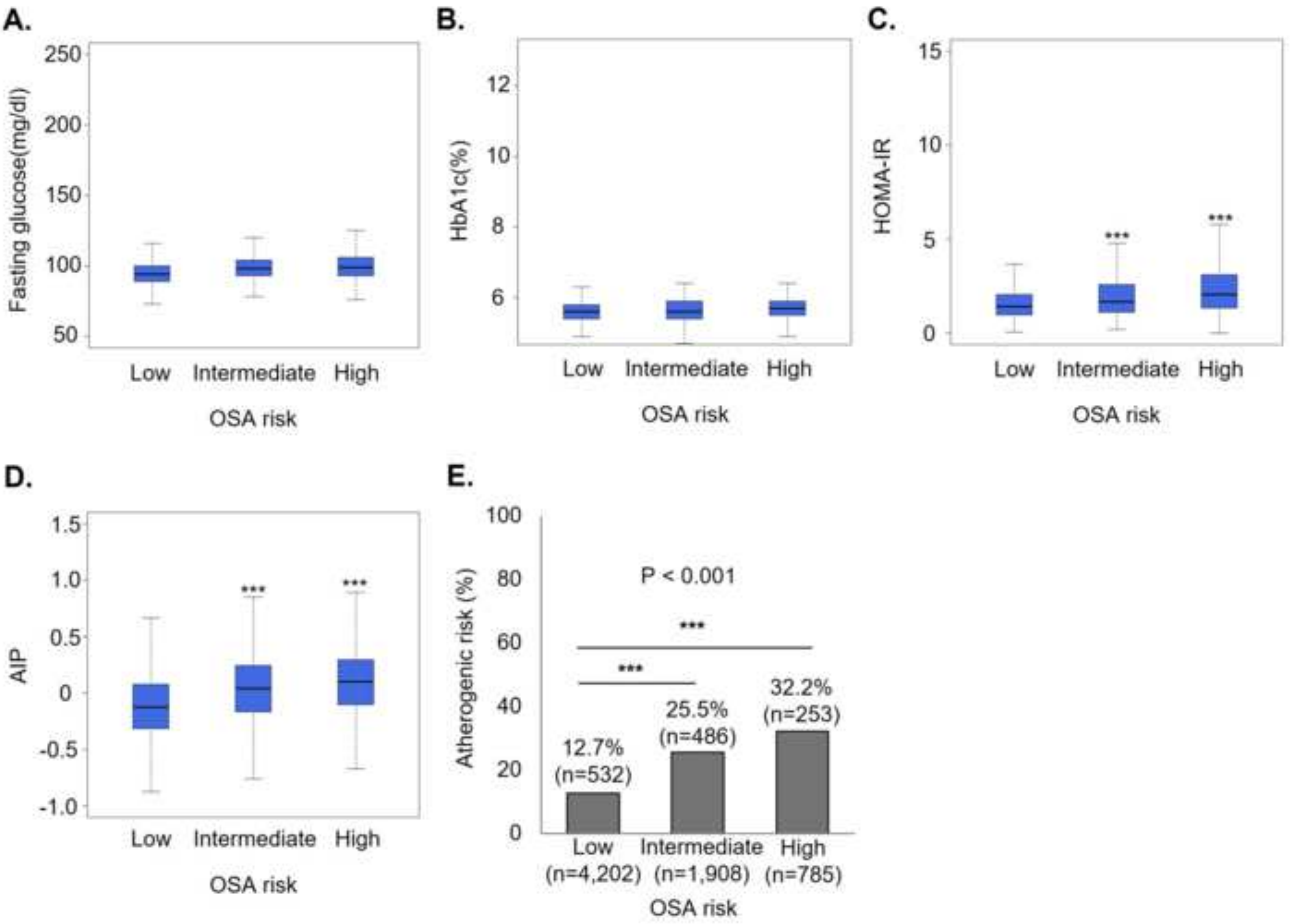

Figure4

Click here to access/download;Figure;FIG4.tiff

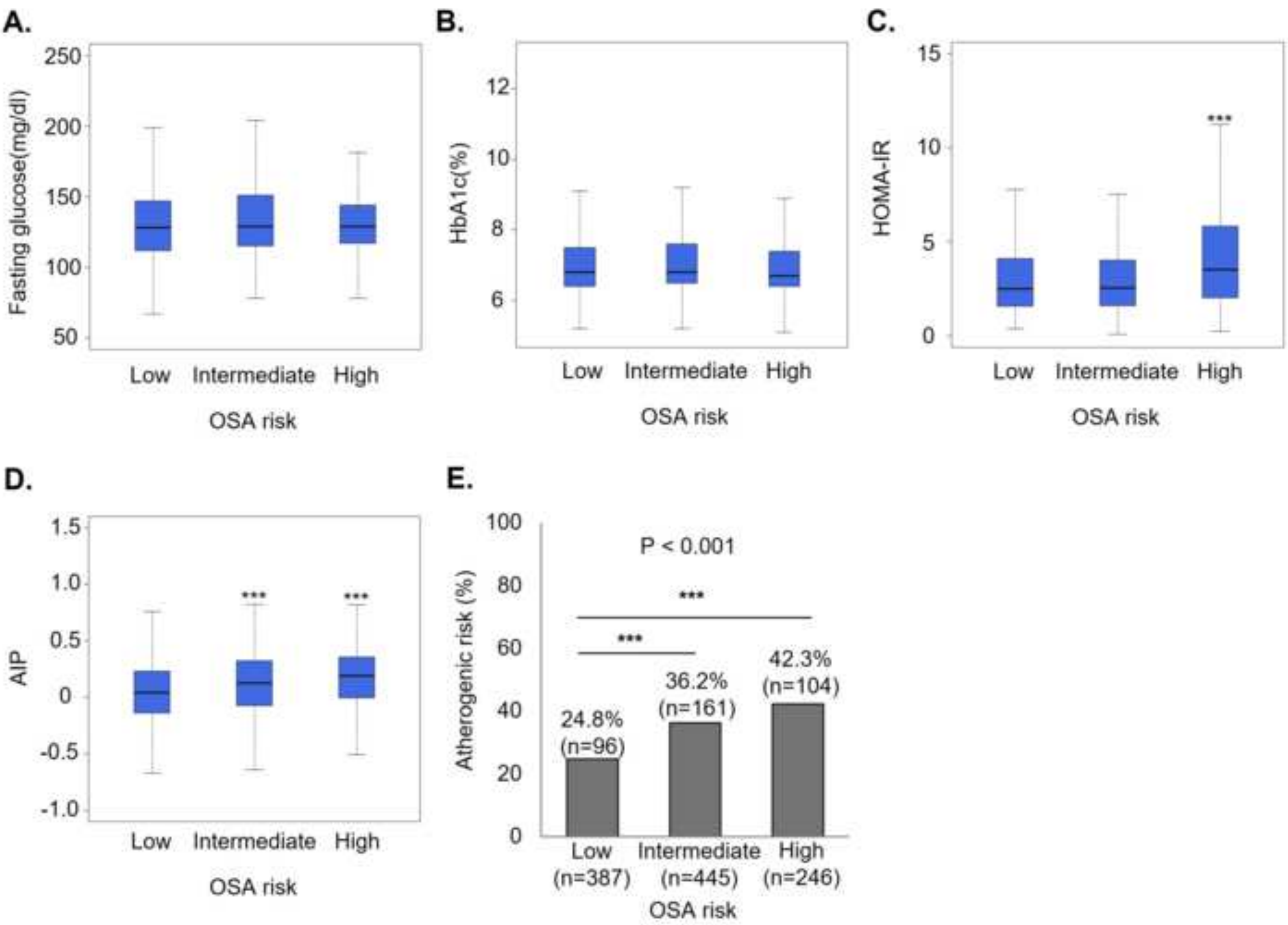

Supplement: Attachment — Submitted filename: PONE-D-24-46140.pdf [file pone.0322897.s001.pdf]
