# Supplementary material for: Association between obstructive sleep apnea risk and atherosclerosis: A nationwide cross-sectional study in the Korean population
Source: PLoS One. 2025 May 8;20(5):e0322897. doi: 10.1371/journal.pone.0322897 (PMC12061421; doi:10.1371/journal.pone.0322897)
Supplement: Attachment — Submitted filename: Response to Reviewers.docx [file pone.0322897.s003.docx]

Reviewer #1: The article is sounds intersting and up to the mark and statistical data is also ok but one revision i want to suggest you which is you used too old references like one is 1981 and some reference are 2001 or before 2015 try to use the references like from 2010 to 2024.. I am recoomending this with minor revision.

Thank you for your comments.

As you suggested, we have removed references published before 2010 and replaced them with more recent studies, ensuring the references from 2010 to 2024. The updated references are recorded in the revised manuscript for your review.

However, we have retained the following pre-2010 references due to their foundational significance in the field: the 2002 study by Kapur, V. et al., which provides a comprehensive analysis of the underestimation of sleep apnea prevalence; the 2008 study by Chung, F. et al., introducing the concept of the STOP-BANG score; and the 2006 study by Dobiásová, M. et al., which presents the concept of AIP.

We appreciate your understanding and thank you for your valuable suggestions.

Reviewer #2: Few modifications have been suggested.
1. Page no. 7, line no. 138-to mention accreditation status of laboratories where tests were conducted
2. Page no. 7, line no. 145-reference is needed
3. line no. 152-is it operational definition?
4. Page no.10, line no. 208-to mention rationality of conducting non-parametric tests

Thank you for your comments.

1. KNHANES provides various standards and regulations to ensure quality control of laboratory tests. This information has been included in the revised manuscript.
2. The definitions of the variables analyzed in this study are referenced from KNHANES. Therefore, KNHANES has been cited as a reference.
3. The definition of aerobic exercise is also referenced from KNHANES. Additionally, this definition aligns with the National Physical Activity Guidelines published by the U.S. Department of Health and Human Services.
4. The rationale for conducting non-parametric tests has been mentioned in the revised manuscript.

We appreciate your valuable suggestions.
